# The history of uveitis: from antiquity to the present day

**DOI:** 10.1038/s41433-024-03519-x

**Published:** 2024-12-04

**Authors:** Nima Ghadiri

**Affiliations:** 1 Department of Ophthalmology, Liverpool University Hospitals NHS Foundation Trust, Liverpool, UK; 2 Department of Eye and Vision Science, University of Liverpool, Liverpool, UK

**Keywords:** Education, Autoimmune diseases, Autoimmune diseases

## Abstract

This comprehensive review traces the historical understanding and treatment of uveitis from ancient civilisations to modern medicine, a history which reflects the evolution of medical understanding and practice in general. Early descriptions of ocular inflammation appear in Egyptian medical papyri from 1700 BCE, with subsequent contributions from Greek and Roman physicians, including Hippocrates and Galen, who provided foundational observations of ocular inflammatory conditions. Medieval scholars in the Middle East, particularly Avicenna, preserved and advanced classical knowledge while recognising the systemic nature of ocular inflammation. The Renaissance and Enlightenment periods brought anatomical precision and the emergence of ophthalmology as a distinct specialty, notably through innovations such as Helmholtz’s ophthalmoscope in 1851. The twentieth century marked a paradigm shift with the evolution of immunological understanding, leading to the recognition of autoimmune mechanisms and genetic predispositions in uveitis. The introduction of corticosteroids mid-century revolutionised treatment, followed by the development of steroid-sparing immunomodulatory agents and, more recently, targeted biological therapies. Contemporary advances in imaging technology and the establishment of international collaborative groups have standardised classification and treatment approaches. This historical perspective demonstrates the progression from empirical observations to precision medicine in uveitis, highlighting the importance of understanding this evolution for advancing future therapeutic strategies.

## Introduction

Uveitis, a condition characterised by inflammation of the uveal tissues, which include the iris, ciliary body, and choroid, is a complex and multifaceted ailment with a diverse range of aetiologies. Causes can range from infections and autoimmune disorders to being of unknown origin.

It is an important contributor to loss of sight around the world, estimated to account for around 10% of visual impairment in the Western world. Each year, there are an estimated 17–52 new cases per 100,000 people, and prevalence ranges from 38–714 per 100,000 people [1].

Throughout history, this condition has posed a quandary for diagnosis and treatment, due to its complexity. However, as medical knowledge and practices have evolved over time, so too has our understanding of uveitis and its systemic implications. By delving into the past, we can gain a deeper insight into this puzzling condition and how it has been viewed in different eras of medicine.

### Early historical descriptions of uveitis

The first known references to inflammation of the eye can be traced back to the ancient civilisations of Egypt, Greece, Rome, Persia, China, and India. These advanced societies of their respective eras recognised the importance of the eye as a vital organ and its susceptibility to various diseases.

In ancient Egypt, medical papyri from approximately 1700 BCE, such as the Edwin Smith Papyrus, include descriptions of ocular conditions that are likely early descriptions of uveitis [2], albeit containing references to ideas and beliefs from even earlier times, dating back as far as 2640 BC.

Physicians who specialised in eye care have been documented as far back as the sixth Egyptian Dynasty (2400 BC). Pepi Ankh Or Iri, the Pharoah’s Royal Oculist, is often recognised as the first documented ophthalmologist in history, although his titles varied from ‘palace eye physician’ to ‘guardian of the anus’ [3]. Physicians of this period believed that many eye diseases were punishments from the divine or caused by external environmental factors, and consequently treated these ailments with herbal remedies and ritualistic practices, such as the use of cleansing to remove toxins from the body. The Ebers papyrus unveiled additional methods of treating various ocular ailments. This ancient text covered a broader range of eye diseases and their potential cures than the Edwin Smith papyrus. Out of the 237 prescribed remedies, 100 were specifically intended for treating eye-related conditions, such as miosis and mydriasis [4].

Although the specific term ‘uveitis’ was not used at the time, the symptoms and characteristics described by these early civilisations align with modern understandings of the disease. In fact, Hippocrates (460–375 BCE) himself, often referred to as the father of modern medicine, wrote about ocular inflammation in his works. He detailed the effects and potential treatments of inflammation in the eye within his ‘Third Book of Endemic Diseases’, describing ophthalmic manifestations of Behçet’s syndrome [5]. Though lacking the anatomical precision required to differentiate uveitis from other causes of ocular redness or vision loss, the Greek luminary’s keen observations and thorough documentation laid the groundwork for his successors.

This included Roman physician-surgeon Galen (129–216 CE), who advanced the understanding of the eye by recognising the roles of inflammation and infection in vision disorders. His was the first mention of the ‘choroid’ and even of choroiditis [6]. Despite his contributions, understanding and treating ocular inflammation remained in its infancy, with treatments geared towards alleviating symptoms rather than tackling root causes.

### Medieval and renaissance advances

In the torpid centuries of the Middle Ages, the study of medicine, including ophthalmic medicine, in Europe was suppressed by religious doctrines that rebuffed scientific exploration. However, in the Middle East, scholars diligently preserved and enriched ancient Greek and Roman medical texts.

For example, Avicenna (Ibn Sina, 980–1037 CE) made groundbreaking contributions to the field in the eleventh century, meticulously documenting various eye diseases, including those now classified as forms of uveitis. His seminal work, ‘*The Canon of Medicine*,’ stressed the vital role of observation and clinical practice, leaving a lasting impression on both Eastern and Western medicine. The Persian polymath recognised ocular inflammation as part of a broader systemic disorder [7], marking an early recognition of the relationship between eye health and overall bodily conditions—a core concept in the study of uveitis.

During the European Renaissance, figures like Andreas Vesalius (1514–1564 CE) and others reinvigorated anatomical study with a scientific approach, further advancing the understanding of ocular structures. Their detailed dissections allowed for more accurate anatomical descriptions of the uvea, improving diagnostic capabilities [8]. It was at this time, that ocular findings in relation to conditions such as Syphilis were identified, with Juan de Vigo identifying and describing the ocular manifestations of the ‘great imitator’ in 1567 [9].

### The enlightenment and early modern period

During the seventeenth and eighteenth centuries, there were notable advancements made in the understanding of the eye and its pathologies. The invention of the microscope by Hans and Zacharias Janssen *circa* 1590, and observations of microbial life through Antony van Leeuwenhoek in 1676 [10] heralded an era whereby ocular tissues were examined more closely. This led to the identification of inflammatory cells within the uvea. As a result, the term ‘uveitis’ became more prevalent in medical writings as doctors worked towards categorising and comprehending the different types of ocular inflammation.

In the nineteenth century, ophthalmology emerged as a distinct medical specialty, further propelling the study of uveitis. Visionaries such as Hermann von Helmholtz, who created the ophthalmoscope in 1851, revolutionised the ability to visually explore the interior of the eye [11]. This groundbreaking device allowed clinicians to directly observe signs of ocular inflammation, leading to more precise diagnoses and a deeper understanding of its clinical manifestations.

The Age of Enlightenment was characterised by scientific rigour and experimentation, and this catalysed significant advances in ophthalmology. François Boissier de Sauvages (1706–1767 CE) and William Mackenzie (1791–1868 CE) were among the first to detail uveitis as a distinct clinical entity, recognising its association with systemic conditions such as tuberculosis and syphilis. Mackenzie’s textbook, *A Practical Treatise on the Diseases of the Eye* (1830), was instrumental in establishing uveitis as a distinct diagnostic category [12].

Indeed, Tuberculosis and Syphilis were generally seen as the primary culprits for Uveitis in the late nineteenth century. Robert Koch’s discovery of *Mycobacterium tuberculosis* in 1882 [13], and its subsequent identification as a cause of uveitis, represented a turning point in linking systemic infections with ocular inflammation. This period also marked the beginning of using immune-suppressing therapies for the treatment of ocular inflammation. However, the precise treatments were unique to their time, and included application of ‘leeches put to the temple’ [14] and mercury [15], alongside dilation of the pupil with belladonna tincture and use of antimony [12].

### Uveitis in the twentieth century: immunology and infectious disease connections

The development of immunology in the twentieth century had a profound impact on the understanding of uveitis, and at the century’s turn, Uhlenhuth [16] explained the phenomenon of sympathetic ophthalmia, detailing how autoimmune responses could be elicited after immunisation with lens antigen. Elschnig subsequently postulated that a uveitogenic trigger was present in the eye [17].

Further research in immunopathology revealed that many cases of uveitis were autoimmune in nature, occurring as part of systemic diseases such as sarcoidosis, Behçet’s disease, and rheumatoid arthritis. Goldmann’s theory of the blood–ocular barrier was crucial in understanding the relationship between eye inflammation and systemic inflammatory diseases [18]. This landmark shed light on how immune responses within the eye could lead to persistent inflammation, even without an external infection present.

However, this early part of the twentieth century also saw some radical methods of treating presumed immune-mediated diseases such as ocular inflammation, including fever therapy. This treatment involved injecting milk into the muscle or administering triple typhoid H antigen intravenously, with the aim of raising the body’s core temperature to around 40 °C. The theory behind this therapy was that the body’s release of corticosteroids during times of high stress could improve its effectiveness. While there were instances of success, the results of this treatment were often unpredictable and even fatal, and reflected the challenges of understanding this disease at the time [19].

Advances in laboratory diagnostics, including serological testing and radiographic imaging, allowed for more accurate differentiation between infectious and non-infectious causes of uveitis.

In the 1960s and 70s, there was a surge of interest in antibody-mediated mechanisms. Researchers believed that immune complex-mediated disease (specifically, type III hypersensitivity reactions) could be responsible for a large portion of the observed diseases [20]. This theory was reinforced by the presence of circulating immune complexes in patients with uveitis, although further examination of uveitis eyes did not support this mechanism. Concurrently, advancements in technology allowed for widespread testing of histocompatibility—the era of the human leukocyte antigen (HLA), and the finding that diseases with ocular complications could be linked to a genetic predisposition [21].

### Uveitis in the twentieth century: unveiling the triggers

The discovery that environment has a role in Uveitis became clear within the twentieth century. For example, within the aetiological detective work carried out to uncover the cause of Behçet’s disease (for which many attempts to find an infective cause had not previously borne fruit). It was noted that the disease is seen more often in the North than the South of Japan [22], though genetic analysis could not explain this observation—and it was postulated that an environmental trigger might be playing a role, for example, concomitant streptococcus infection.

The understanding of immunogenetics was also progressing in this era, with a greater understanding of how specific HLA types, such as HLA-B27, can activate a predisposition to uveitis, particularly in the context of spondyloarthropathies. Birdshot chorioretinopathy was found to be closely linked to HLA-A29 [23], Behçet’s disease to HLA-B51 [24], and Vogt–Koyanagi–Harada syndrome [25] to MT3 (HLA-DR53), and new associations are being discovered to this day.

### Uveitis in the twentieth century: treatments

The introduction of corticosteroids [26] in the mid-twentieth century provided a powerful tool for managing ocular inflammation. Gordon’s discovery was groundbreaking and promised to revolutionise management, and this breakthrough brought hope to patients suffering from inflammatory disorders, despite the potential for long-term adverse effects. As years passed, the side effects of chronic use of corticosteroids became well-documented and widely published. These included electrolyte imbalances, myopathy, osteoporosis, tendon rupture, nausea, gastrointestinal issues, poor wound healing, easy bruising, neurological disturbances, menstrual irregularities, and weight gain [27]. Ocular effects were also observed, such as an increased risk for cataracts, glaucoma, central serous retinopathy, and activation of herpes simplex virus [28]. While corticosteroids effectively suppressed many inflammatory diseases, their significant side effect profile made them unacceptable for prolonged usage.

As a result, corticosteroid-sparing immunomodulatory agents were developed. The first group of drugs developed for immunosuppression were the alkylating agents, such as cyclophosphamide and chlorambucil, which inhibit lymphoid proliferation. Cyclophosphamide was created from a chemical warfare agent in the 1950s and was first used to treat uveitis [29]. Chlorambucil, developed in the 1950s, was originally used for malignant lymphoma before being used to treat uveitis [30]. Another category of immunosuppressants, antimetabolites such as  methotrexate and azathioprine, inhibit key enzymatic reactions necessary for cell reproduction [31]. Methotrexate was first used for acute leukaemia in children before being utilised for ocular inflammatory disorders. Azathioprine was developed for transplant patients and autoimmune diseases in the 1960s, but was later used to treat ocular immune-mediated disorders.

In the 1970s, noncytotoxic immunosuppressives were introduced for treating autoimmune diseases [32]. Cyclosporin A was derived from fungi in 1983 and led to further investigations for future therapies due to its adverse effects. Tacrolimus, and Sirolimus, were discovered in the 1980s and 1990s, respectively [33]. Mycophenolate mofetil emerged in 1995 and was initially used for preventing organ transplant rejection but later also for uveitis treatment [34]. Many of these immunosuppressive treatments remain firm within the therapeutic arsenal.

### Modern advances in uveitis and the era of collaboration

The late twentieth and early twenty-first centuries subsequently witnessed an explosion of knowledge regarding the genetic and molecular underpinnings of uveitis. But it was the revolutions in imaging technology which slowly took over as driver for discovery. Technological advances, including optical coherence tomography [35], fluorescein angiography [36], and anterior segment optical coherence tomography [37], revolutionised the ability to visualise intraocular inflammation in unprecedented detail, allowing for earlier detection and more precise management of uveitis.

The recognition of early intervention as a critical factor in preventing vision loss and improving long-term outcomes in uveitis has gained increasing prominence, supported by numerous studies. Nevertheless, significant challenges persisted in the diagnosis and management of uveitis, prompting the establishment of collaborative organisations. The International Uveitis Study Group, founded in 1978 [38], and the International Ocular Inflammation Society, established in 1989, played pivotal roles in advancing evidence-based approaches and fostering consensus among clinicians. These efforts culminated in the Standardization of Uveitis Nomenclature (SUN) initiative, which has been working towards international consensus on the classification criteria for uveitis [SUN] since 2004 [39].

Steady further understanding of the immunopathology in Uveitis has allowed for the development of more bespoke therapies, targeting specific cytokines or cell surface receptors for more precise immunosuppression. Biologic therapies, such as tumour necrosis factor inhibitors and interleukin inhibitors, have provided new treatment avenues, particularly for patients with refractory non-infectious uveitis. Muromonab was the first of its kind, developed in the mid-1980s for treating glucocorticoid-resistant rejection [40]. In the late 1990s, drugs like etanercept were created to target specific autoimmune disorders like rheumatoid arthritis. Other biologics, including abatacept, adalimumab, daclizumab, infliximab, anakinra, rituximab and tocilizumab soon followed [41]. Treatment modalities such as intravenous immunoglobulin and interferon-γ also emerged. These therapies, alongside refined surgical techniques, have improved outcomes and reduced complications for patients with uveitis.

The future of uveitis treatment will involve a combination of advanced biologics, gene therapies, precision medicine and AI-driven tools, all of which will enhance both preventative and therapeutic approaches. The ongoing emphasis on early detection and multidisciplinary collaboration will be key to improving patient outcomes and reducing the burden of this potentially sight-threatening disease.

## Conclusion

The history of uveitis illustrates the continually evolving trajectory of medical knowledge, originating from ancient descriptions of ocular diseases and progressing to the contemporary understanding of immunology, genetics and systemic health. Recognising this historical context is essential for advancing future care, as it underscores the longstanding recognition of the intricate relationship between ocular and systemic well-being.

As our comprehension of the immune system and the complexities of ocular inflammation deepens, the future of uveitis management is poised for significant advancement. Ongoing research efforts continue to refine diagnostic methods and therapeutic strategies, offering promising prospects for improved patient outcomes in the future.

## References

[1] Wakefield D, Chang JH. Epidemiology of uveitis. Int Ophthalmol Clin. 2005;45:1–13. 10.1097/01.iio.0000155938.83083.94. 10.1097/01.iio.0000155938.83083.94 15791154

[2] Krause AC. Ancient Egyptian ophthalmology. Bull Inst Hist Med. 1933;1:258–76.

[3] Breasted J. The Edwin Smith surgical papyrus. Chicago: University of Chicago Press; 1930.

[4] Ebbell B. Die altagyptische Chirurgie. Die chirurgischen Abschnitte des Papyrus E. Smith and Papyrus Ebers. Oslo: Dybwad; 1939.

[5] Feigenbaum A. Description of Behçet’s syndrome in the Hippocratic third book of endemic diseases. Br J Ophthalmol. 1956;40:355–7. 10.1136/bjo.40.6.355. 13355940 10.1136/bjo.40.6.355 PMC509495

[6] Jackson E. Review of literature of chronic uveitis. Read in a symposium on chronic uveitis at the second annual meeting of The Association for Research in Ophthalmology. Philadelphia; 1931.

[7] Avicenna A. Ghanoon dar teb. Soroosh. Tehran; 1988. p. 244–51.

[8] De Laey J. The eye of Vesalius. Acta Ophthalmol. 2011;89:293–300. 10.1111/j.1755-3768.2009.01679.x 20584000 10.1111/j.1755-3768.2009.01679.x

[9] Lemoine AN. Ocular manifestations of congenital syphilis and treatment by induced hyperpyrexia. Trans Am Ophthalmol Soc. 1934;32:522–54. 16693026 PMC1315491

[10] Barow ME. The development of the microscope. Bios. 1934;5:157–64.

[11] McMullen WH. The evolution of the ophthalmoscope. Br J Ophthalmol. 1917;1:593–9. 10.1136/bjo.1.10.593. 18167712 10.1136/bjo.1.10.593 PMC513361

[12] MacKenzie W. A practical treatise on the diseases of the eye. London: Longman, Rees, Orme, Brown & Green; 1830. p. 422–57.

[13] Koch R. Die atiologie der tuberkulose. Berl Klin Wochenschr. 1882;19:221–2.

[14] Knapp H. A Case of traumatic dislocation of the Iris under the unbroken conjunctiva - eye damaged but preserved, Typical sympathetic ophthalmia in the other eye. Trans Am Ophthalmol Soc. 1893;6:513–23. 16691866 PMC1327621

[15] Kollock CW. Use of mercury in traumatic irido-choroiditis. Trans Am Ophthalmol Soc. 1896;7:602–6. 16691900 PMC1327090

[16] Uhlenhuth PT. Zur lehre yon unterscheidung verschiedener eiweissarten mit hilfe spezifischer sera. In: Festschrift zum 60 geburtstag von Robert Koch. Jena, German Democratic Republic: Fischer; 1903. p. 49–74.

[17] Elschnig A. Studien zur sympatischen ophthalmis. Die antigeue wirkung des augenpigmentes. Albrecht yon Graefes Arch Ophthalmol. 1910;76:509–46.

[18] Goldmann EE. Vitalbarfung am Zentralnervensystem. Abhandl Konigl Preuss Akad Wiss 1913;1:1–60.

[19] Heath WD. Why did fever therapy work? Arch Ophthalmol. 1985;103:1457. 10.1001/archopht.1985.01050100025008. 4051847 10.1001/archopht.1985.01050100025008

[20] O’Connor GR. Factors related to the initiation and recurrences of uveitis, XL Edward Jackson memorial lecture. Am J Ophthalmol. 1983;96:577–99. 6139024 10.1016/s0002-9394(14)73415-4

[21] Brewerton DA, Caffrey M, Nicholls A, Walters D, James DC. Acute anterior uveitis and HL-A 27. Lancet. 1973;302:994–6. 4127279 10.1016/s0140-6736(73)91090-8

[22] Nussenblatt RB, Palestine AG. Uveitis: fundamentals and clinical practice. Chicago: Year Book Medical Publishers; 1989.

[23] Nussenblatt RB, Mittal KK, Ryan S, Green WR, Maumenee AE. Birdshot retinochoroidopathy associated with HLA-A29 antigen and immune responsiveness to retinal S-antigen. Am J Ophthalmol. 1982;94:147–58. 6956239 10.1016/0002-9394(82)90069-1

[24] Chajek-Shaul T, Pisanty S, Knobler H, Matzner Y, Glick M, Ron N, et al. HLA-B51 may serve as an immunogenetic marker for a subgroup of patients with Behçet’s syndrome. Am J Med. 1987;83:666–72. 3314492 10.1016/0002-9343(87)90896-5

[25] Davis JL, Mittal KK, Freidlin V, Mellow SR, Optican DC, Palestine AG, et al. HLA associations and ancestry in Vogt-Koyanagi-Harada disease and sympathetic ophthalmia. Ophthalmology. 1990;97:1137–42. 2234843 10.1016/s0161-6420(90)32446-6

[26] Gordon D. Prednisone and prednisolone in ocular inflammatory disease. Am J Ophthalmol. 1956;41:593–600. 13302360

[27] Stanbury RM, Graham EM. Systemic corticosteroid therapy—side effects and their management. Br J Ophthalmol. 1998;82:704–8. 9797677 10.1136/bjo.82.6.704 PMC1722622

[28] Renfro L, Snow JS. Ocular effects of topical and systemic steroids. Dermatol Clin. 1992;10:505–12. 1617809

[29] Buckley CE, Gills JP. Cyclophosphamide therapy of peripheral uveitis. Arch Intern Med. 1969;124:29–35. 5791483

[30] Dinning WJ, Perkins ES. Immunosuppressives in uveitis. A preliminary report of experience with chlorambucil. Br J Ophthalmol. 1975;59:397. 1081881 10.1136/bjo.59.8.397 PMC1017383

[31] Andrasch RH, Pirofsky B, Burns RP. Immunosuppressive therapy for severe chronic uveitis. Arch Ophthalmol. 1978;96:247–51. 629670 10.1001/archopht.1978.03910050115002

[32] Hall BM, Tiller DJ, Hardie I, Mahony J, Mathew T, Thatcher G, et al. Comparison of three immunosuppressive regimens in cadaver renal transplantation: long-term cyclosporine, short-term cyclosporine followed by azathioprine and prednisolone, and azathioprine and prednisolone without cyclosporine. N. Engl J Med. 1988;318:1499–507. 3285215 10.1056/NEJM198806093182304

[33] Kelly PA, Burckart GJ, Venkataramanan R. Tacrolimus: a new immunosuppressive agent. Am J health-Syst Pharm. 1995;52:1521–35. 7552894 10.1093/ajhp/52.14.1521

[34] Larkin G, Lightman S. Mycophenolate mofetil: a useful immunosuppressive in inflammatory eye disease. Ophthalmology. 1999;106:370–4. 9951492 10.1016/S0161-6420(99)90078-7

[35] Huang D, Swanson EA, Lin CP, Schuman JS, Stinson WG, Chang W, et al. Optical coherence tomography. Science. 1991;254:1178–81. 1957169 10.1126/science.1957169 PMC4638169

[36] Laatikainen L, Kohner EM. Fluorescein angiography and its prognostic significance in central retinal vein occlusion. Br J Ophthalmol. 1976;60:411–8. 952814 10.1136/bjo.60.6.411 PMC1017520

[37] Hoerauf H, Wirbelauer C, Scholz C, Engelhardt R, Koch P, Laqua H, et al. Slit-lamp-adapted optical coherence tomography of the anterior segment. Graefe’s Arch Clin Exp Ophthalmol. 2000;238:8–18. 10664046 10.1007/s004170050002

[38] Saari KM. The International Uveitis Study Group. Acta Ophthalmol. 1984;62:21–8. 10.1111/j.1755-3768.1984.tb08470.x 6326463

[39] Jabs DA. Standardization of Uveitis Nomenclature (“SUN”), Global, 2004-2021. Inter-university Consortium for Political and Social Research [distributor]. 2024. 10.3886/ICPSR38665.v1.

[40] Johnston A, Holt DW. Therapeutic drug monitoring of immunosuppressant drugs. Br J Clin Pharmacol. 1999;47:339. 10233195 10.1046/j.1365-2125.1999.00911.x PMC2014241

[41] Reimold AM. TNFa as therapeutic target: new drugs, more applications. Curr Drug Targets Inflamm Allergy. 2002;1:377–92. 14561184 10.2174/1568010023344535

